# ACE2 Receptor and TMPRSS2 Protein Expression Patterns in the Human Brainstem Reveal Anatomical Regions Potentially Vulnerable to SARS-CoV-2 Infection

**DOI:** 10.1021/acschemneuro.3c00101

**Published:** 2023-05-12

**Authors:** Aron Emmi, Aleksandar Tushevski, Alessandro Sinigaglia, Silvia Barbon, Michele Sandre, Elena Stocco, Veronica Macchi, Angelo Antonini, Luisa Barzon, Andrea Porzionato, Raffaele De Caro

**Affiliations:** † Institute of Human Anatomy, Department of Neuroscience, University of Padova, 35121 Padova, Italy; ‡ Movement Disorders Unit, Padova University Hospital, 35121 Padova, Italy; § Center for Neurodegenerative Disease Research (CESNE), University of Padova, 35121 Padova, Italy; ∥ Department of Molecular Medicine, University of Padova, 35121 Padova, Italy; ⊥ Department of Cardio-Thoraco-Vascular Sciences and Public Health, University of Padova, 35121 Padova, Italy

**Keywords:** ACE2, TMPRSS2, SARS-CoV-2, COVID-19, neuropathology, brain

## Abstract

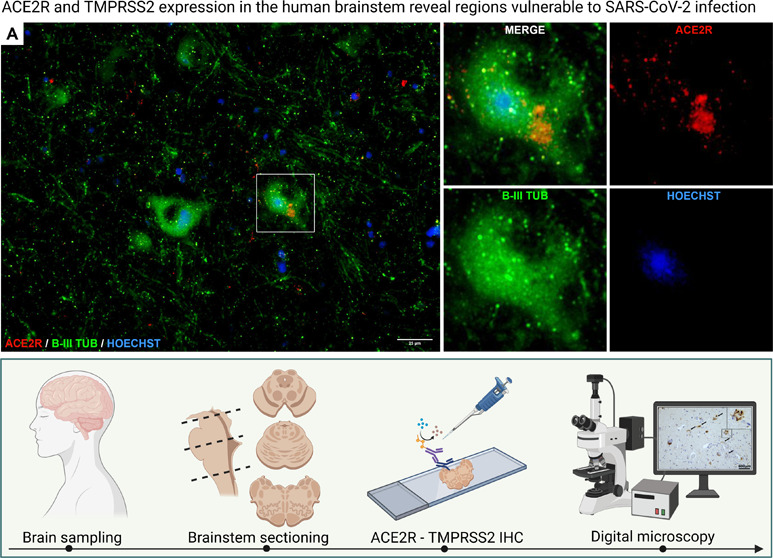

Angiotensin-converting enzyme 2 receptor (ACE2R) is a transmembrane protein expressed in various tissues throughout the body that plays a key role in the regulation of blood pressure. Recently, ACE2R has gained significant attention due to its involvement in the pathogenesis of COVID-19, the disease caused by the Severe Acute Respiratory Syndrome CoronaVirus 2 (SARS-CoV-2). While ACE2 receptors serve as entry points for the novel coronavirus, Transmembrane Serine Protease 2 (TMPRSS2), an enzyme located on the cell membrane, is required for SARS-CoV-2 S protein priming. Even though numerous studies have assessed the effects of COVID-19 on the brain, very little information is available concerning the distribution of ACE2R and TMPRSS2 in the human brain, with particular regard to their topographical expression in the brainstem. In this study, we investigated the expression of ACE2R and TMPRSS2 in the brainstem of 18 adult subjects who died due to pneumonia/respiratory insufficiency. Our findings indicate that ACE2R and TMPRSS2 are expressed in neuronal and glial cells of the brainstem, particularly at the level of the vagal nuclei of the medulla and the midbrain tegmentum, thus confirming the expression and anatomical localization of these proteins within specific human brainstem nuclei. Furthermore, our findings help to define anatomically susceptible regions to SARS-CoV-2 infection in the brainstem, advancing knowledge on the neuropathological underpinnings of neurological manifestations in COVID-19.

## Introduction

Severe acute respiratory syndrome coronavirus 2 (SARS-CoV-2) is a recently discovered strain of Coronavirus that was first reported in China, in the city of Wuhan,^1^ as a viral cause of new outbreaks of pneumonia. The World Health Organization (WHO) named the infectious disease caused by SARS-CoV-2 as Coronavirus Disease 2019 (COVID-19), with the main symptoms not only composed of respiratory distress, fever, fatigue, and cough but also characterized by frequent neurological manifestations.

The constellation of neurological symptoms reported following SARS-CoV-2 infection include headache, dizziness, delirium, encephalopathy, ataxia, seizures, increased stroke risk, and encephalitis and also suggest peripheral nervous system involvement (vision and smell impairments, sympathoactivation, etc.).^2−5^ Neuropathological findings in COVID-19 patients include hypoxic–ischemic damage, neuroinflammation with prominent microgliosis and lympho-monocytic infiltrates, as well as instances suggesting SARS-CoV-2 neurotropism. We have previously demonstrated that SARS-CoV-2 viral proteins and genomic sequences can be detected in specific brainstem nuclei of a subset of COVID-19 decedents and that microglial cells present a topographically defined pattern of distribution within the brainstem while also displaying a more severe inflammatory phenotype compared to pneumonia subjects.^5^ However, there is very little information concerning the anatomical distribution of proteins that can mediate viral binding and cell entry of the novel coronavirus, despite numerous studies addressing the direct and indirect neuropathological sequelae of SARS-CoV-2 infection.

Viral entry into host cells is mainly mediated by the S envelope protein, which is composed of two subunits named S1 and S2.^6^ Virus attachment to the target cells involves S1 and the host angiotensin-converting enzyme 2 (ACE2) receptor. The following steps are allowed by the cellular proteins cathepsin L and transmembrane protease serine 2 (TMPRSS2) that, acting on the S1/S2 complex, lead to the exposure of a fusion peptide belonging to the S2 subunit (Harrison et al.^6^). SARS-CoV-2 entry is further facilitated by TMPRSS2 by proteolytically cleaving and activating the glycoproteins of the viral envelope.^7,35^

SARS-CoV-2 is supposed to directly access the central nervous system and potentially infect the resident neuronal cells expressing ACE2R by either exploiting the blood–brain barrier or via retro-axonal dissemination through the olfactory nerves and the olfactory bulb^8^ or through the glossopharyngeal and vagus nerve.^9^

Furthermore, the binding of the S protein to ACE2R is known to lead to a drop in soluble ACE2, a homologue of angiotensin-converting enzyme (ACE). In the central nervous system, ACE mediates neuroinflammation, neurodegeneration, and neurotoxicity and is involved in several neurological disorders, with ACE2 counteracting the effects of ACE. Hence, aside from direct damage induced by the virus and indirect consequences mediated by systemic inflammation and the ongoing cytokine storm, neurotoxic and neurodegenerative effects modulated by ACE2 can be blunted by inactivation of ACE2R following SARS-CoV-2 S protein binding.^10^

Interestingly, studies have reported differences in the expression of ACE2R levels between young and older adults, suggesting ACE2R expression increases with age.^11^ This finding is also correlated with higher morbidity and mortality rates from COVID-19 in the elderly population.^12^ However, despite the relevant role played by these proteins in both health and disease, studies on ACE2R distribution and expression throughout the human CNS are limited,^13−17^ and very little information is available concerning the brainstem, despite prominent involvement of this structure in COVID-19; in particular, Hill et al.^14^ revealed prominent ACE2R expression at the level of the pons by means of ELISA analysis; Lukiw et al.^38^ also detected high levels of ACE2R expression in the pons and medulla oblongata in human subjects. Conversely, Lindskog et al.^17^ performed immunohistochemical analyses on COVID-19 and normal brain samples but did not identify ACE2R expression in brainstem neurons, with the choroid plexus representing the most prominent site of immunoreactivity. Hence, further research is warranted to determine ACE2R expression in the brainstem and, more precisely, in individual brainstem nuclei.

In the present study, we aim to define the topographic expression of ACE2R and TMPRSS2 throughout the human brainstem, with particular regard to brainstem nuclei involved in COVID-19. For this purpose, we have selected a cohort of 18 adult subjects (8 female, 10 male, age 72 ± 12 years) who died due to pneumonia or respiratory insufficiency. Clinical information for this cohort is available in Table S1, as reported previously.^5^

## Results

### Medulla Oblongata

**Figure 1:**
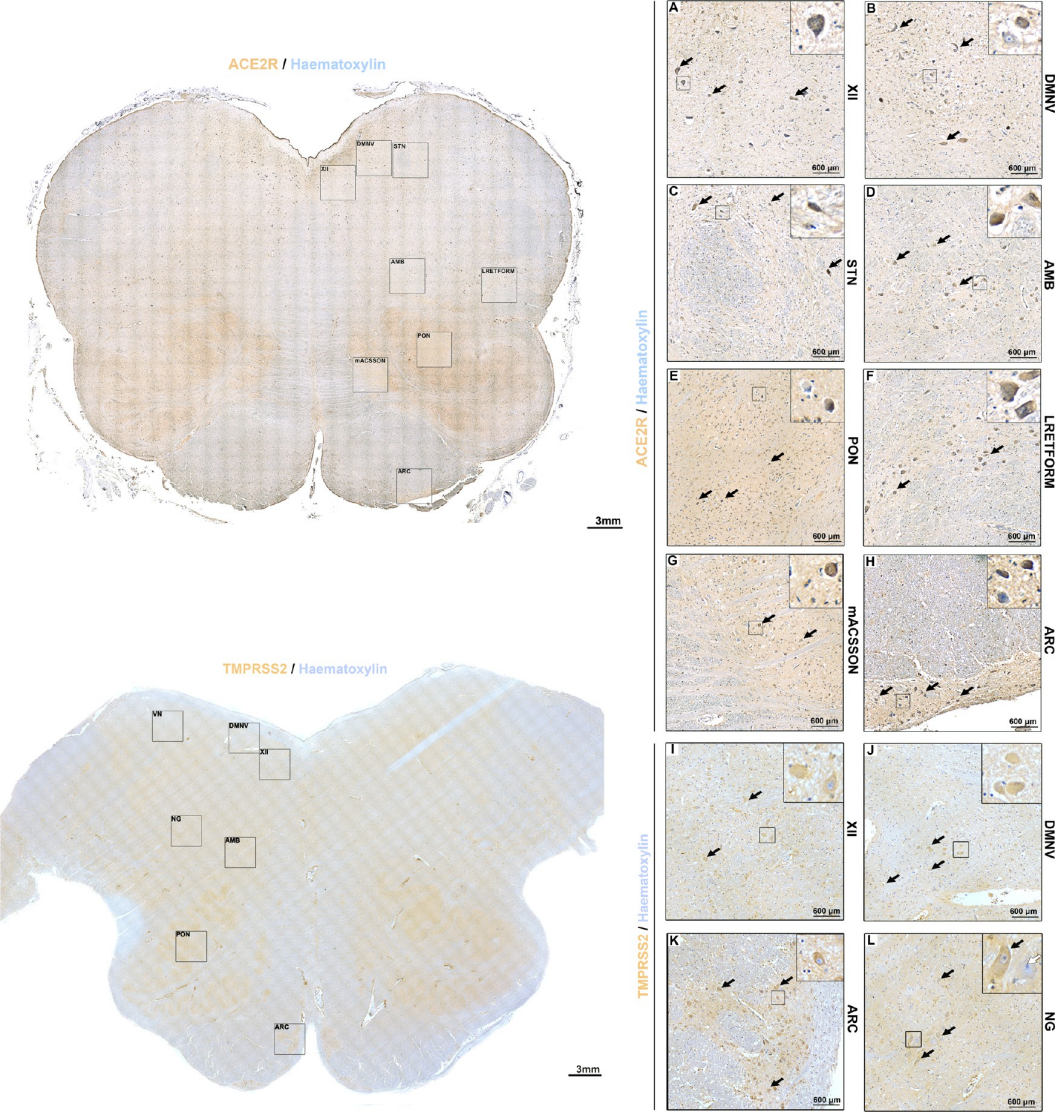
(A–H) Immunoperoxidase staining for ACE2R in the human medulla reveals a specific topographical expression that is comparable to TMPRSS2 staining (I–L).

At the level of the medulla oblongata, ACE2R displayed mild to moderate immunoreactivity. ACE2R immunoreactivity was more prominent in the medullary tegmentum compared to the ventral medulla. In particular, the hypoglossal nucleus (Figure 1A), dorsal motor nucleus of the vagus (Figure 1B), and solitary tract nucleus (Figure 1C) displayed mild neuropil immunoreactivity and moderate neuronal cytoplasmatic reactivity. At the level of the solitary tract, glial immunoreactivity was also present (Figure 1C). A similar pattern of reactivity was also found for the nucleus ambiguus and the lateral reticular formation of the medulla (Figure 1D,F). White matter bundles such as the anterolateral system, the spinal trigeminal route, and the medial lemniscus were not reactive. In the ventral medulla, moderate neuropil immunoreactivity was found at the level of the olivary complex (Figure 1E,G). While most neurons of the olivary complex were not immunoreactive, sporadic mildly immunoreactive neurons were also detected. The arcuate nucleus (Figure 1H), on the other hand, displayed marked ACE2R neuronal cytoplasmic reactivity.

TMPRSS2 displayed mild to moderate immunoreactivity at the level of the medulla oblongata. TMPRSS2 immunoreactivity was more prominent in the medullary tegmentum compared to the ventral medulla. In particular, the hypoglossal nucleus, dorsal motor nucleus of the vagus, and solitary tract nucleus (Figure 1I–J) displayed mild neuropil immunoreactivity and moderate to marked neuronal cytoplasmatic reactivity.

A similar pattern of reactivity was also found for the nucleus ambiguus and the lateral reticular formation of the medulla with predominantly moderate neuronal cytoplasmatic TMPRSS2 immunoreactivity. At the level of the olivary complex in the ventral medulla, moderate neuropil immunoreactivity was found; however, in the arcuate nucleus (Figure 1K), marked neuronal cytoplasmatic immunoreactivity was observed. As for ACE2R, most neurons at the olivary complex were not TMPRSS2-immunoreactive.

### Pons

At the level of the pons, ACE2R showed mild to moderate immunoreactivity.

**Figure 2:**
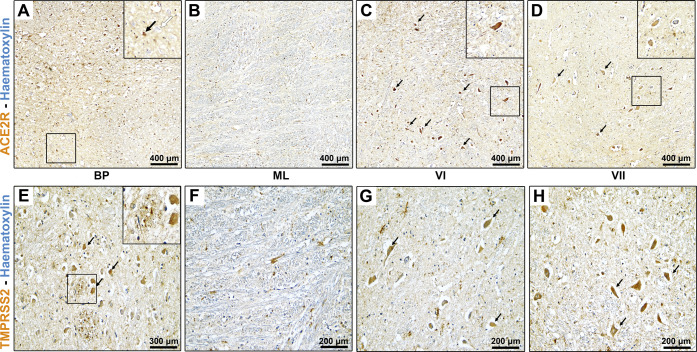
(A–D) Immunoperoxidase staining for ACE2R in the human pons reveals predominant expression in cranial nerve nuclei of the dorsal pons. (E–H) Similar pattern of TMPRSS2 immunoreactivity is observed.

In particular, immunoreactivity for ACE2R was moderate to mild at the level of basilar pons (Figure 2A), with both neuronal cytoplasmic reactivity and glial immunoreactivity, as well as moderate neuropil immunoreactivity. The medial lemniscus showed no neuronal cytoplasmic immunoreactivity for ACE2R; however, moderate glial immunoreactivity was observed (Figure 2B).

Stronger immunoreactivity for ACE2R was detected at the level of the dorsal parts of the pons, with moderate to marked neural cytoplasmic immunoreactivity within the facial and abducens nuclei (Figure 2C,D). The overall neuropil generally showed moderate immunoreactivity.

A similar pattern of staining was found for TMPRSS2 in the pons.

At the level of the basilar pons (Figure 2E), moderate neuronal cytoplasmic reactivity and glial immunoreactivity, as well as mild neuropil immunoreactivity, were detected for TMPRSS2. The medial lemniscus showed no neuronal cytoplasmic immunoreactivity for TMPRSS2; however, mild glial immunoreactivity was observed (Figure 2F).

Stronger immunoreactivity for TMPRSS2 was detected in the dorsal parts of the pons, with moderate to marked neural cytoplasmic immunoreactivity within the facial and abducens nuclei (Figure 2G,H), similar to ACE2R. The overall neuropil was mildly immunoreactive.

### Midbrain

**Figure 3:**
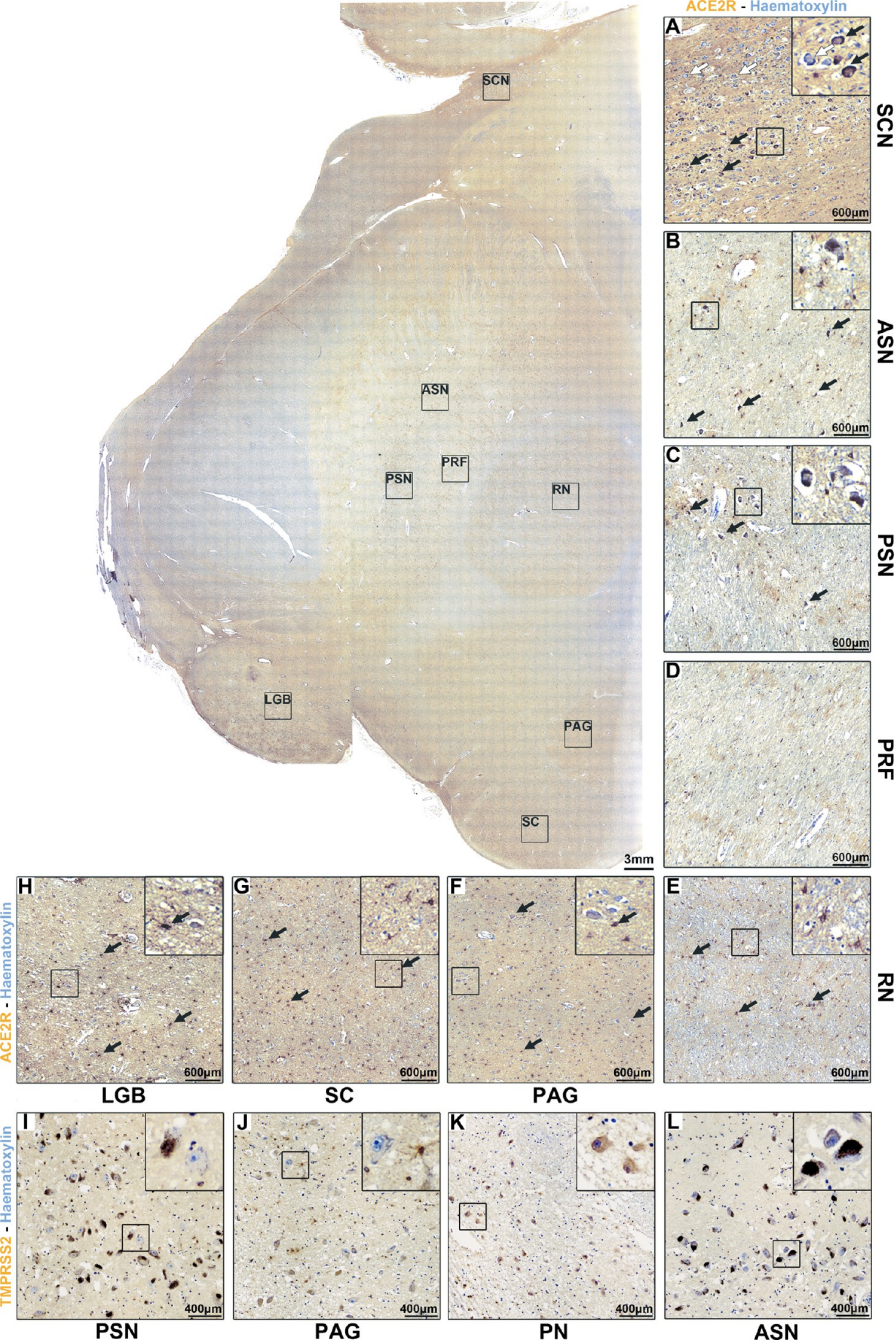
(A–H) Immunoperoxdiase staining for ACE2R in the human midbrain reveals region-specific expression. (I–L) Immunoperoxdiase staining for TMPRSS2 in the human midbrain reveals region-specific expression patterns comparable to ACE2R.

The midbrain displayed moderate to marked ACE2R immunoreactivity (Figure 3).

**Figure 4:**
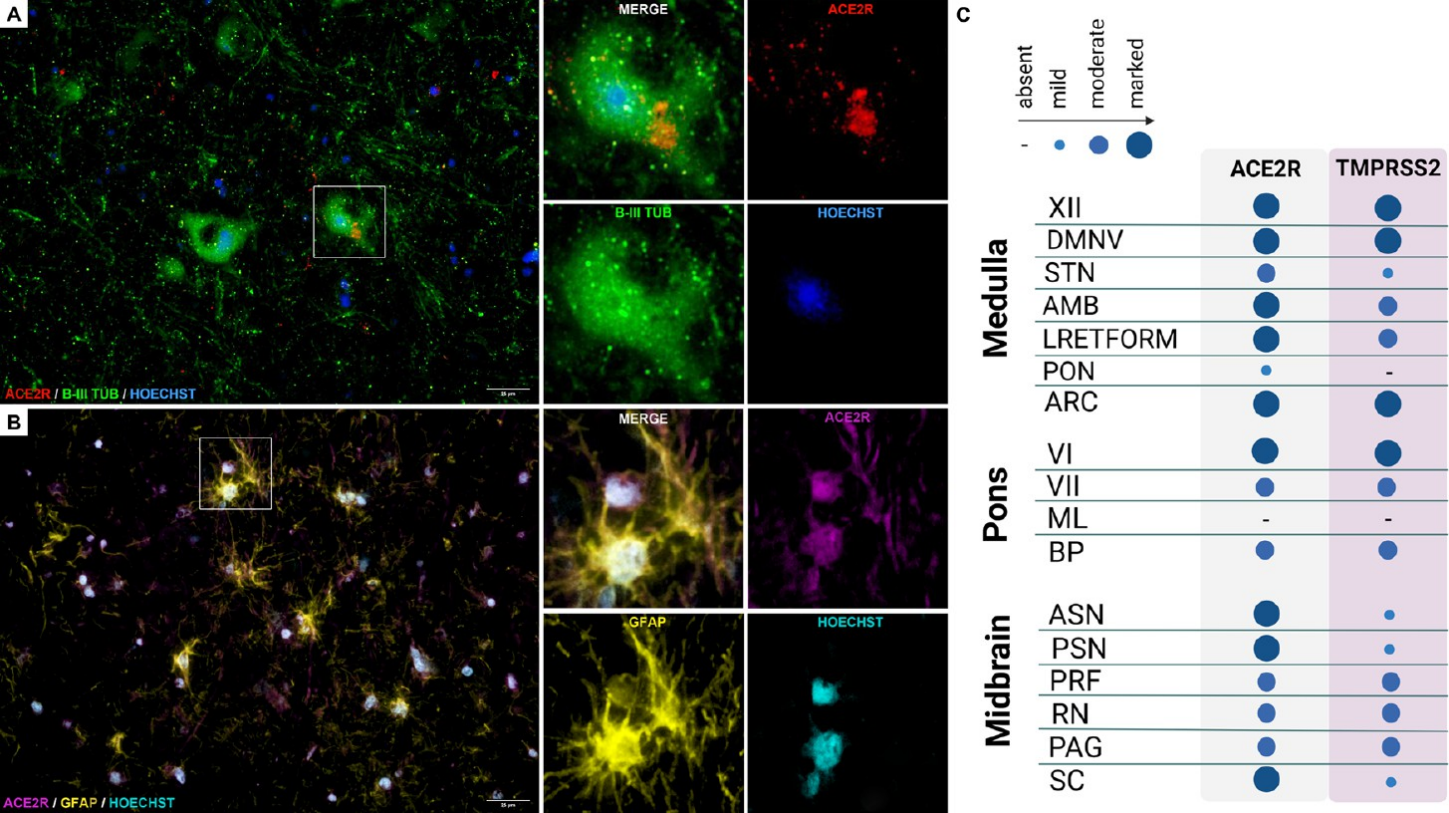
(A) Immunofluorescent staining for ACE2R (red) and pan-neuronal marker β-III-tubulin (green) reveals neuronal cytoplasmic expression of ACE2R in the dorsal medulla. (B) Similar pattern of ACE2R reactivity (magenta) is found for GFAP-positive (yellow) astrocytes. (C) Median values of the four-tiered semiquantitative morphometrical evaluation of ACE2R and TMPRSS2 immunoreactivity throughout the brainstem: −, negative; ●, mild; ●, moderate; ●, marked.

ACE2R immunoreactivity was highly prominent in both the mesencephalic tegmentum and ventral mesencephalon, mostly in astrocytes (as seen in immunofluorescent staining, Figure 4). In particular, the substantia nigra showed mild to undetectable neural cytoplasmic immunoreactivity to ACE2R in both the anterior and posterior parts (Figure 3B,C), as well as moderate to marked glial ACE2R immunoreactivity. The same pattern of ACE2R immunoreactivity was evident in the lateral geniculate body (Figure 3H). The prerubral field and the red nucleus showed moderate glial and neuropil ACE2R immunoreactivity (Figure D–E). Furthermore, the periaqueductal gray had moderate neuronal cytoplasmatic and glial reactivity (Figure 3F), while the superior colliculus showed marked glial ACE2R immunoreactivity. Finally, some control samples included the suprachiasmatic nucleus, which showed marked neuronal cytoplasmatic ACE2R immunoreactivity (Figure 3G).

As for TMPRSS2 in the midbrain, neuronal cytoplasmic immunoreactivity was mildly detectable only at the level of the periaqueductal gray and red nucleus (Figure 3J,K), while little-to-no significant cytoplasmic immunoreactivity was found in the substantia nigra (Figure 3I,L).

### Identification of ACE2R Expressing Cells in the Human Brainstem

The reactivity patterns provided following immunofluorescence staining of brainstem sections showed clear colocalization of ACE2R with β-III-tubulin positive structures, a pan-neuronal marker, indicating ACE2R expression within neurons (Figure 4A).

Additional immunofluorescence staining highlighted the colocalization of ACE2R with GFAP, confirming the presence of this receptor also on astrocytes (Figure 4B). Oligodendroglial immunoreactivity was not detectable.

The semiquantitative estimation of ACE2R and TMPRSS2 immunoreactivity are reported in the panel of Figure 4C.

## Discussion

ACE2 receptor is known to be widely expressed throughout various tissues and organs of the human body.^18^ The results provided by the present study demonstrate the expression of ACE2R and TMPRSS2 protein also in the human brainstem.

ACE2R and TMPRSS2 protein immunohistochemistry reveals a specific topographical organization throughout the brainstem, with more evident neuronal and neuropilar immunoreactivity being detected at the level of the dorsal medulla, showing moderate to marked neuronal immunoreactivity at the level of the dorsal motor nucleus of the vagus, the hypoglossal nucleus, the medullary reticular formation, and the nucleus ambiguus. Interestingly, we also detected marked expression of ACE2R and TMPRSS2 in the neurons of the arcuate nucleus. This group of neurons is thought to carry on chemosensory functions and modulate the activity of respiratory centers, potentially influencing breathing patterns,^19^ even though the role of this nucleus in respiratory circuits is debated. The immunoreactivity for ACE2R and TMPRSS2 was less evident at the level of the pons, with sparse immunoreactive neurons and glia in the basal pons and in the pontine tegmentum.

Immunoreactivity for ACE2R and TMPRSS2 was also found in the midbrain at the level of the substantia nigra and periaqueductal gray but was less pronounced compared to the medulla.

Overall, while the expression of ACE2R and TMPRSS2 was mostly moderate in the human brainstem, immunoreactive neurons and glial cells were clearly detected in several anatomical loci. On the other hand, Lindskog et al.^17^ recently evaluated the expression of ACE2R in the human brain but did not detect significant neuronal or glial immunoreactivity in the brainstem. Various aspects need to be taken into account to explain differences in our studies: cohort size, post-mortem interval, differences in fixation, antigen retrieval methods, and antibodies employed. Nevertheless, their findings are particularly valuable due to the inclusion of COVID-19 subjects and the evaluation of bulk RNA expression of ACE2 in other brain regions. Furthermore, our cohort is characterized mostly by elderly subjects with relevant medical comorbidities who died due to pneumonia or respiratory insufficiency. Although not comparable to healthy subjects of the general population, this cohort more realistically represents the elderly population with relevant medical comorbidities that is particularly susceptible to SARS-CoV-2 infection and vulnerable to severe or fatal COVID-19 manifestations. We have not detected significant differences in ACE2R and TMPRSS2 expression in response to brainstem gliosis and ischemic injury, as displayed by Table S1 and Figure S1, aside from a positive correlation (*r* = 0.65, *p* = 0.01) between brainstem hypoxic–ischemic damage and nucleus ambiguus ACE2R expression. Conversely, we have found statistically significant correlations between age and hypertension (*r* = 0.62, *p* = 0.006) as well as hypertension and hypoxic–ischemic damage of the brainstem (*r* = 0.59, *p* = 0.01), as expected for a cohort of elderly subjects with relevant medical comorbidities and similarly to previously published COVID-19 cohorts.^5^

Qualitative staining differences related to age were also detected, with older subjects generally presenting more pronounced immunoreactivity for the investigated proteins. Yet, due to sample size limitations, correlation between brainstem ACE2R expression levels and age was not statistically significant. Further studies are required to evaluate ACE2R and TMPRSS2 expression in younger subjects and in populations without other medical comorbidities.

The detection of anatomically susceptible regions for SARS-CoV-2 entry is particularly relevant, given recent evidence strongly suggesting viral neurotropism. Moreover, other coronaviruses, such as SARS-CoV and MERS-CoV, are known to be able to infect the brainstem in both humans and animal models of the disease and particularly the dorsal motor nucleus of the vagus, solitary tract nucleus, and nucleus ambiguus by retrograde axonal transport through the vagus nerve, so that an analogue pattern of neuroinvasion for SARS-CoV-2 has been suggested. Matschke et al. detected SARS-CoV-2 viral proteins in cranial nerves IX-X and in the brainstem of COVID-19 decedents.^9^ Similarly, we have detected SARS-CoV-2 viral proteins and genomic sequences in the vagal nuclei of the medulla, as well as the substantia nigra of the midbrain.^5^ Moreover, we have previously documented SARS-CoV-2 infection of the carotid body, which is connected to the medulla oblongata through the carotid sinus nerve, a branch of the glossopharyngeal cranial nerve (IX);^36,37^ this is further supported by Vitale-Cross et al.,^13^ who detected ACE2R expression in the human glossopharyngeal and vagus nerve terminals. The findings of this study further confirm the susceptibility of these brainstem nuclei to viral infection due to the expression of ACE2R and TMPRSS2.

Interestingly, previous studies have demonstrated ACE2 downregulation by SARS-CoV-2 spike protein, which in turn leads to an overactivation of the Angiotensin II axis. Angiotensin II is known to lead to the development of macrophage activation syndrome and promote the cytokine storm in COVID-19, while in the CNS, Angiotensin II is also known to cause increased secretion of vasopressin and sympathetic activation.^20−22^

The downregulation of ACE2R by SARS-CoV-2 is achieved through at least two known host proteases, the TMPRSS2 and disintegrin metalloproteinase domain-containing protein 17 (ADAM17), also known as tumor necrosis factor α converting enzyme, which downregulates ACE2 by scattering it through the circulatory system.^23,24^

In addition, ACE2 is a known essential modulator of the renin–angiotensin–aldosterone system,^25−27^ which has been found to be dysfunctional following SARS-CoV-2 infection.^28^ Interestingly, studies have also demonstrated that ACE2 expression increases with aging, which in turn might explain the predominance of severe morbidity and mortality in older subjects, as well as more predominant neurological manifestations and acceleration of prior neurodegenerative diseases.^29^

In conclusion, our findings help to define anatomically susceptible regions of the brainstem to coronavirus infection, with particular regard to SARS-CoV-2. Furthermore, due to the involvement of ACE2R in the renin–angiotensin–aldosterone system, as well as Angiotensin II-related pathways, the detection of these proteins in the parasympathetic and cardio-respiratory nuclei of the brainstem further enforces the role of sympathetic activation.

## Methods

### Subjects and Inclusion Criteria

The brains of 18 subjects deriving from the Body Donation Program of the University of Padova (Italy) were employed for the study.^30^ The neuropathological examination and the clinical data of these subjects were previously reported in Emmi et al., 2023,^5^ serving as a control group for the aforementioned study.

The inclusion criteria required (1a) negative testing for SARS-CoV-2 infection confirmed through molecular testing of rhino-pharyngeal swabs or (1b) date of death prior to the 2019 COVID-19 pandemic and (2) high-quality brain tissue samples available for histopathological and immunohistochemical analysis, determined by post-mortem interval (PMI) ≤5 days, fixation time ≤3 weeks, absence of tissue maceration, and adequate formalin penetration in the tissue.

### Sampling and Fixation Procedures

Following autopsy (mean PMI = 3 days), the brains were sampled and immersion-fixed in 4% formalin solution with an average fixation time of 2–3 weeks.

The brains were then sectioned according to established neuropathological examination protocols. The brainstem was sectioned at the level of the rostral extremity of the midbrain and extensively sampled in its whole cranio-caudal extent.

Prior to paraffin embedding, a slow dehydration and clearing protocol was performed in order to preserve epitope binding.

### Histochemical and Immunohistochemical Staining

Slides were cut by means of a calibrated sliding microtome with a fixed thickness of 5 μm. Routine histopathological evaluation was performed by haematoxylin and eosin staining.

Immunoperoxidase staining was performed manually according to previously established protocols^31−34^ following antigen retrieval performed on de-paraffinized tissue sections using Dako EnVision PTLink station according to the manufacturer’s recommendations. Antibodies for ACE2 receptor protein (Rabbit anti-Human polyclonal, citrate buffer HIER, dilution 1:2000, Abcam, code number: ab15348) and TMPRSS2 protein (Rabbit anti-Human monoclonal, citrate buffer HIER, dilution 1:2500, Abcam, code number: ab242384) were employed. Antibodies were validated on positive control tissues (human kidney samples) and by antibody omission, revealing specificity.

### Immunofluorescent Staining and Confocal Microscopy

Fluorescent immunohistochemistry was performed manually following antigen retrieval performed on de-paraffinized tissue sections using Dako EnVision PTLink station according to the manufacturer’s recommendations. Following antigen retrieval, autofluorescence was quenched with a 50 mM NH_4_Cl solution for 10 min. The sections were then treated with a permeabilization and blocking solution (15% vol/vol goat serum, 2% wt/vol BSA, 0.25% wt/vol gelatin, 0.2% wt/vol glycine in PBS) containing 0.5% Triton X-100 for 120 min before primary antibody incubation. The following antibodies were employed: ACE2 receptor protein (#ab15348; dilution 1:500); β-III-tubulin (#T8578; dilution 1:300); GFAP (polyclonal Mouse anti-Human, proteinase K enzymatic antigen retrieval, dilution 1:250, DAKO Omnis, code number: GA524).

Primary antibodies were diluted in a blocking solution and incubated at 4 °C overnight. Alexa-Fluor plus 488 Goat anti-Mouse secondary antibody (code number: A32723) and Alexa-Fluor plus 568 anti-Rabbit secondary antibody (code number: A-11011) were diluted 1:200 in blocking solution as above and incubated for 60 min at room temperature. To further avoid background signal and tissue autofluorescence, slides were incubated for 10 min in 0.5% Sudan Black B solution in 70% ethanol at room temperature and abundantly washed with PBS, followed by Hoechst 33258 nuclear staining (Invitrogen, dilution: 1:10 000 in PBS) for 10 min. Slides were mounted and coverslipped with Mowiol solution (prepared with Mowiol 4–88 reagent, Merck Millipore, code number: 475904–100GM). Confocal immunofluorescence z-stack images were acquired on a Leica SP5 confocal laser scanning microscope using a HC PL FLUOTAR 20×/0.50 Dry or HCX PL APO lambda blue 40×/1.40 Oil objectives. Images were acquired at a 16-bit intensity resolution over 2048 × 2048 pixels. Z-stacks images were converted into digital maximum intensity z-projections, processed, and analyzed using ImageJ software.

### Histopathological and Morphometrical Evaluation

Slides were examined by two independent histopathologists and morphologists blind to subject conditions. Disagreements were resolved by consensus. The degree ACE2R and TMPRSS2 expression was classified using a four-tiered semiquantitative approach (0, absent; 1, mild; 2, moderate; 3, marked) for each evaluated section, assessed by means of digitally assisted immunoreactivity quantification by two independent evaluators. Disagreements were resolved by consensus. The scores for each region of interest (ROI) are reported in Tables S2 and S3. The median of the semiquantitative scores was used to represent average expression across subjects.

## Data Availability

All data are available upon request to the corresponding author.

## Supporting Information Available

The clinical and demographic data of the study cohort (Table S1), the raw semiquantitative scores for each investigated ROI (Table S2 for ACE2R expression and Table S3 for TMPRSS2 expression), and the Spearman correlation matrix used to evaluate correlations between clinical and demographic information and ACE2R expression scores (Figure S1) (PDF)

## Notes

All of the material used in this study was obtained through the body donation program of the Institute of Human Anatomy–University of Padova, in accordance with Italian Law 10/2020. All procedures were performed according to the declaration of Helsinki.

## Abbreviations

ACE2R: angiotensin-converting enzyme 2 receptor
AMB: nucleus ambiguus
ARC: arcuate nucleus
ASN: anterior substantia nigra
BP: basilar pons
CNS: central nervous system
DMNV: dorsal motor nucleus of the vagus
LGB: lateral geniculate body
LRETFORM: lateral reticular formation
mACSSON: medial accessory olivary nucleus
ML: medial lemniscus
PAG: periaqueductal gray
PON: olivary nucleus proprium
PRF: prerubral field
PSN: posterior substantia nigra
RN: red nucleus
SARS-CoV-2: severe acute respiratory syndrome coronavirus 2
SC: superior colliculus
SCN: suprachiasmatic nucleus
STN: solitary tract nucleus
TMPRSS2: transmembrane serine protease 2
VI: abducens cranial nerve nucleus
VII: facial cranial nerve nucleus
XII: hypoglossal nucleus
