# Supplementary material for: ACE2 Receptor and TMPRSS2 Protein Expression Patterns in the Human Brainstem Reveal Anatomical Regions Potentially Vulnerable to SARS-CoV-2 Infection
Source: ACS Chem Neurosci. 2023 May 12;14(11):2089–97. doi: 10.1021/acschemneuro.3c00101 (PMC10228702; doi:10.1021/acschemneuro.3c00101)
Supplement: Supplementary file 1 — cn3c00101_si_001.pdf [file cn3c00101_si_001.pdf]

Supporting information

ACE2 Receptor and TMPRSS2 protein expression patterns in the Human Brainstem reveal anatomical regions potentially vulnerable to SARS-CoV-2 infection

Aron Emmi§[1,2,3], Aleksandar Tushevski§[1], Alessandro Sinigaglia[4], Silvia Barbon[1], Michele Sandre[2,3], Elena Stocco[1,5], Veronica Macchi[1], Angelo Antonini[2,3], Luisa Barzon[4], Andrea Porzionato[1,3]*, Raffaele De Caro[1,3]

[1]Institute of Human Anatomy, Department of Neuroscience, University of Padova, 35121, Padova, Italy

[2]Movement Disorders Unit, Padova University Hospital, 35121, Padova, Italy

[3]Center for Neurodegenerative Disease Research (CESNE), University of Padova, 35121, Padova, Italy

[4]Department of Molecular Medicine, University of Padova, 35121, Padova, Italy

[5]Department of Cardio-Thoraco-Vascular Sciences and Public Health, University of Padova, 35121, Padova, Italy

§ these authors contributed equally

* Corresponding Author: Prof. Andrea Porzionato

email: andrea.porzionato@unipd.it

Department of Neuroscience

University of Padova, Italy.

ID	Age	Sex	Hospitalization (days)	Hypertension	PMI	Antemortem Head CT	Neurological signs	Neuropathological evaluation	Brainstem Hypoxic damage	Microthrombosis (NON-CNS)	Cause of Death
#1	83	M	10	Y	5	Cerebral atrophy, Chronic ischaemic vascular disease	Cognitive decline	Mixed dementia with AD neuropathological changes ad chronic ischaemic vascular disease	Moderate	No	Pneumonia, respiratory insufficiency, ischaemic heart disease
#2	74	M	2	Y	4	Vascular calcification, ischaemic heart disease	NA	Chronic ischaemic vascular disease	Mild	No	Ischaemic heart disease.
#3	40	M	1	N	4	No signs	No signs	No detectable microscopical changes	No detectable microscopical changes	No	Haemorragic Shock
#4	79	F	31	Y	3	Cerebral atrophy, Chronic ischaemic vascular disease	Cognitive decline, Alzheimer type	AD neuropathological changes, CAA, ischemic vascular disease	Mild	No	Pentalobar pneumonia, respiratory insufficiency
#5	62	M	22	Y	5	NA	NA	Diffuse hypoxic/ischaemic damage	Mild	No	Pneumonia, respiratory insufficiency
#6	76	M	15	Y	6	NA	NA	Ischaemic vascular disease, diffuse hypoxic/ischaemic damage	Mild	No	Pneumonia, chronic ischaemic vascular disease
#7	75	M	12	Y	4	NA	NA	Diffuse hypoxic/ischaemic damage	Mild	No	Pneumonia, ischaemic heart disease
#8	78	F	8	Y	3	No	NA	Diffuse hypoxic/ischaemic damage	Mild	No	Pneumonia, ischaemic heart disease
#9	71	F	40	Y	3	NA	NA	Diffuse hypoxic/ischaemic damage	Moderate to severe	No	Acute respiratory failure, septic shock, peritonitis
#10	46	F	15	N	4	No signs	No signs	Diffuse hypoxic/ischaemic damage	No detectable microscopical changes	No	Respiratory insufficiency, multiorgan failure, cervical neoplasia
#11	75	F	20	Y	5	Chronic Ischaemic Vascular disease, Cerebral atrophy	NA	Vascular dementia, ischaemic vascular disease, diffuse hypoxic/ischaemic damage	Moderate	No	Pneumonia, acute respiratory failure, candidosis
#12	80	F	8	Y	4	Cerebral atrophy	Cognitive decline, Alzheimer type	AD neuropathological changes, diffuse hypoxic/ischaemic damage	Moderate	No	Pentalobar pneumonia, respiratory insufficiency
#13	81	F	NA	Y	3	NA	NA	Diffuse hypoxic/ischaemic damage	Mild	No	Pneumonia, acute respiratory failure
#14	63	F	NA	N	3	No signs	No signs	Diffuse hypoxic/ischaemic damage	Mild	No	Ischaemic heart disease
#15	70	M	38	Y	5	Chronic ischaemic vascular disease	NA	Ischaemic vascular disease, diffuse hypoxic/ischaemic damage	Moderate	No	Bilateral pneumonia, respiratory insufficiency.
#16	81	M	10	Y	4	Cerebral atrophy	Cognitive decline, Alzheimer type	Mixed AD neuropathological changes and Lewy Body pathology, diffuse hypoxic/ischaemic damage	Moderate	No	Pneumonia, respiratory insufficiency
#17	75	M	58	Y	6	Cerebral atrophy	NA	Vascular dementia, ischaemic vascular disease, diffuse hypoxic/ischaemic damage	Moderate	No	Pneumonia, respiratory insufficiency.
#18	87	M	30	Y	6	Cerebral atrophy	Cognitive decline	AD neuropathological changes, diffuse hypoxic/ischaemic damage	Moderate	No	Pneumonia, multiorgan failure.

Table S1. Clinical and Demographic data of the study cohort.

	Medulla							Pons				Midbrain					
**Subject**	**XII**	**DMNV**	**STN**	**AMB**	**LRETF.**	**PON**	**ARC**	**VI**	**VII**	**ML**	**BP**	**ASN**	**PSN**	**PRF**	**RN**	**PAG**	**IC/SC**
#1	3	3	2	3	2	1	3	3	3	0	2	3	3	2	NA	1	3
#2	3	3	1	1	2	1	NA	3	2	0	1	3	3	2	NA	1	3
#3	2	2	1	1	1	0	NA	2	1	0	1	3	2	1	2	2	3
#4	2	3	2	2	3	1	3	3	2	0	2	2	2	2	2	2	2
#5	3	3	2	3	3	1	3	3	2	0	2	2	3	2	2	3	3
#6	2	2	2	3	3	2	NA	3	1	0	2	3	3	3	2	2	3
#7	3	3	1	3	3	1	3	3	1	0	3	3	3	3	2	1	3
#8	2	3	2	3	3	2	2	2	2	0	2	3	2	2	2	1	3
#9	NA	NA	NA	NA	NA	NA	NA	2	2	0	2	2	3	3	1	2	2
#10	3	2	2	2	1	1	NA	2	2	0	1	2	3	1	NA	2	3
#11	3	3	2	3	3	1	2	3	1	0	1	2	3	1	2	1	3
#12	2	3	2	3	3	1	3	2	2	0	2	3	3	1	2	1	3
#13	2	3	2	2	3	1	3	3	2	0	2	3	3	2	1	2	3
#14	2	3	2	3	3	1	NA	2	1	0	1	3	3	2	1	2	3
#15	3	2	1	3	3	1	NA	2	2	0	2	3	3	2	2	2	3
#16	3	2	2	3	3	1	3	2	2	0	2	3	2	1	NA	2	3
#17	2	2	2	3	2	1	2	3	2	0	2	3	3	2	NA	2	3
#18	3	2	2	3	2	1	NA	2	2	0	2	3	3	1	2	2	3
**Median**	3	3	2	3	3	1	3	2,5	2	0	2	3	3	2	2	2	3

Table S2. Semiquantitative scores of ACE2R expression levels in anatomical regions of interest (ROI) in the medulla, pons and midbrain for each subject. 0, Absent; 1, Mild; 2, Moderate; 3, Marked; NA, Not available (not sampled, not included in section or excluded due to artifact).

	Medulla							Pons				Midbrain					
**ID**	**XII**	**DMNV**	**STN**	**AMB**	**LRETF.**	**PON**	**ARC**	**VI**	**VII**	**ML**	**BP**	**ASN**	**PSN**	**PRF**	**RN**	**PAG**	**IC/SC**
#1	3	3	2	2	2	0	2	3	3	0	2	0	2	1	NA	1	1
#2	2	3	1	1	2	0	NA	2	2	0	2	1	1	2	NA	1	1
#3	3	2	1	1	1	0	NA	2	1	0	1	1	1	1	2	2	2
#4	2	2	2	2	3	0	3	2	2	0	2	1	2	1	2	2	1
#5	3	3	1	2	3	0	3	3	2	0	2	1	1	2	2	3	1
#6	2	2	1	2	2	0	NA	3	1	0	2	2	1	2	2	2	2
#7	3	3	1	2	3	0	3	3	1	0	3	1	1	2	2	1	2
#8	3	3	2	1	2	0	2	2	2	0	2	1	2	2	2	1	1
#9	NA	NA	NA	NA	NA	NA	NA	2	2	0	2	1	1	1	1	2	1
#10	2	2	1	1	1	0	NA	2	2	0	2	1	1	1	NA	2	1
#11	3	3	1	2	1	0	2	3	1	0	1	1	1	1	2	1	1
#12	2	2	1	2	2	0	3	2	2	0	2	1	1	2	2	1	1
#13	3	3	1	2	2	0	3	3	2	0	2	1	2	2	1	2	1
#14	3	3	2	2	2	0	NA	2	1	0	1	1	2	2	1	2	1
#15	3	2	1	2	2	0	NA	3	2	0	1	1	1	2	2	2	1
#16	2	3	2	2	2	0	3	3	2	0	2	1	1	2	NA	2	1
#17	2	3	1	2	2	0	2	3	2	0	2	1	1	2	NA	2	1
#18	2	3	2	1	2	0	NA	3	2	0	2	2	1	1	2	2	1
**Median**	3	3	1	2	2	0	3	3	2	0	2	1	1	2	2	2	1

Table S3. Semiquantitative scores of TMPRSS2 expression levels in anatomical regions of interest (ROI) in the medulla, pons and midbrain for each subject. 0, Absent; 1, Mild; 2, Moderate; 3, Marked; NA, Not available (not sampled, not included in section or excluded due to artifact).

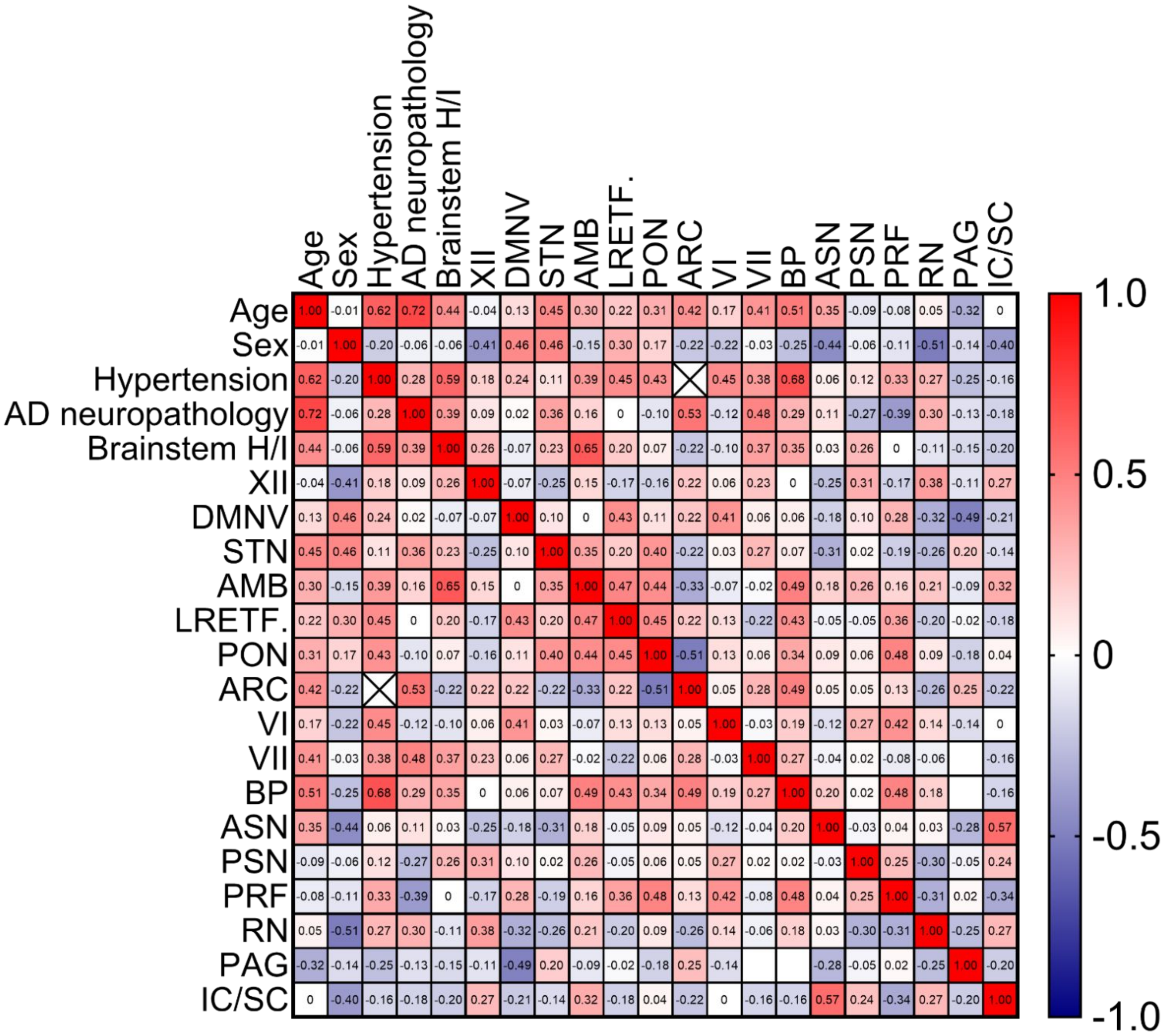

Figure S1. Spearmann Correlation matrix for subject clinical data and semiquantitative scores of ACE2R expression in the brainstem.
